# Pharmacological Properties of the Medical Maggot: A Novel Therapy Overview

**DOI:** 10.1155/2018/4934890

**Published:** 2018-05-03

**Authors:** Litao Yan, Jin Chu, Mingshu Li, Xianfeng Wang, Junwei Zong, Xueyang Zhang, Mingzhi Song, Shouyu Wang

**Affiliations:** ^1^Department of Orthopaedics, The First Affiliated Hospital of Dalian Medical University, Dalian, Liaoning, China; ^2^Department of Ophthalmology, The First Affiliated Hospital of Dalian Medical University, Dalian, Liaoning, China; ^3^Department of Endocrinology, The First Affiliated Hospital of Dalian Medical University, Dalian, Liaoning, China; ^4^Department of Orthopaedics, The Third Affiliated Hospital of Dalian Medical University, Jinpu New District, Liaoning, China

## Abstract

In the last decade, maggot has been hailed as the miraculous “medicinal maggot” for its diverse properties, including antimicrobial, antibiofilm, anti-inflammatory, and wound healing activities. The fact that maggots show so many beneficial properties has increased the interest in these tiny larvae dramatically. Whilst there is relatively abundant clinical evidence to demonstrate the success of maggots as debridement agents, not so much emphasis has been placed on the basic science evidence, which was a combination of physical and biochemical actions. This review differs from those earlier works in that it is undertaken to provide an update of the latest scientific basis published on maggot, particularly active ingredients within maggot excretions/secretions (ES). Further investigations should focus on the isolation, identification, recombination, transgenosis, and mass production of the beneficial molecules within maggots.

## 1. Introduction

Maggot is the larvae of* Lucilia sericata*, belonging to the family Calliphoridae within the order Diptera, which is one of the traditional Chinese herbal medicines. In ancient China, it was described as “quality of cold, flavour, invigorating spleen, heat to eliminate rickets” in the Compendium of Materia Medica and was widely used in folk medicine to treat infantile malnutrition.

Worldwide, live maggot has been used to clean wounds by degrading, liquefying, and ingesting only devitalized/necrotic tissues for centuries. This biotherapy is well known as the maggot debridement therapy (MDT) [1]. Zacharias and Jones were the first that applied maggots to the wounds during the American Civil War. However, it was not until 2004 that the permission was granted for the use of medical maggots by Food and Drug Administration (FDA) [2].

The MDT has experienced a cycle of “clinics--laboratory evidences--clinics”. Put simply, the maggot was first found to be beneficial in clinic and then studied in the laboratory. Finally, the species is further used for more precise clinical applications. It is shown promising for therapeutic application with less expense and adverse effect, compared with conventional therapeutic methods. Though there is a growing acceptance of clinical applications of the maggot, along with extensive clinical trials to demonstrate the success of MDT, scientific laboratory evidences are still not enough to reveal the biochemical mechanisms underlying the beneficial effects of the maggot. At present, the hot research mainly focuses on the antibacterial effects. The maggot actually has other diverse natural properties, such as favorable effects on anti-inflammatory, angiogenesis, extracellular matrix remodelling as well as fibroblast migration/proliferation.

Here, we collect the latest basic research data published on maggot in recent 20 years, particularly active ingredients of maggot ES, and provide convincing molecular evidence to prove the decisive role of maggots in biotherapy.

## 2. Antibacterial Activity

Wound clearing is the most important stage of MDT, which means removing necrotic and contaminated tissues. In response to bacterial challenges, maggot was found to ingest and kill* E. coli* when it passed through the midgut using confocal microscopy [3]. There was still another suggestion of antibacterial mechanism which was called simple mechanical irrigation by ingesting liquefied necrotic tissues [4].

Long before in 1935, Simmons had revealed the presence of an effective bactericide in maggot ES, which exhibited a potent and rapid disinfection action. The maggot ES consisted of salivary gland secretions and faecal waste products [5, 6].

Numerous investigations have since focused on antibacterial activity in maggot ES against both Gram-positive and Gram-negative bacteria. However, there were many contrary conclusions. For example, Thomas et al. confirmed the antibacterial activity of secretions against a range of bacteria, including* Streptococcus A and B, Staphylococcus aureus, Pseudomonas*, and even methicillin resistant* S. aureus* (MRSA) in a preliminary laboratory study. No evidence of inhibition was shown against neither* Enterococcus *nor* E. coli *or* Proteus* [7]. But afterwards, Barnes et al. provided a contradictory result that maggot ES was more potent against* E. coli* than both* S. aureus* and* P. aeruginosa. *They believed that multiple factors such as the number of maggots, bacterial species, and type of assay could influence its antibacterial potency and thus a standard method to quantify antibacterial activity should be established to make the result consistent and comparable [8]. Interestingly, it was observed in clinic that MDT was a more effective strategy to manage Gram-positive bacteria (e.g.,* S. aureus*) than Gram-negative bacteria (e.g.,* P. aeruginosa*). The authors indicated that a sufficient number of maggots were needed not only for a larger wound, but also to exert an activity against Gram-negative bacteria [9].

Long time ago, it was shown that the maggot ES contained kinds of alkaline compounds (e.g., ammonium carbonate, calcium, allantoin, and urea) that inhibited bacterial growth by rising pH in wounds, as well as providing the optimum environment for proteolytic enzyme activity. Over the past two decades, many researchers have focused on identifying and isolating antimicrobial molecules produced by maggots. Such investigations are driven partly by the dilemma of microorganisms resistance (e.g., MRSA) [10].

On a further experiment, Bexfield et al. discovered that maggot ES exhibited potent and antibacterial activity against MRSA in vitro. Two molecular mass fractions (<500 Dalton (Da) and 0.5–10 kDa) with thermal stability and protease resistance were partially isolated using ultrafiltration techniques [11]. The authors carried out an exhaustive study on the antibacterial activity of the <500 Da fraction against a range of bacteria. Distinct morphological changes were observed in* Bacillus cereus* and* E. coli* [12]. The <500 Da fraction was accurately determined as the empirical formula C_10_H_16_N_6_O_9_ and the molecule was patented and registered as a novel antibiotic, Seraticin [13]. On the basis of above experiment, three categories of low molecular weight compounds with antibacterial activity against* Micrococcus luteus* and* P. aeruginosa* were also isolated from the maggot, including p-hydroxyphenylacetic acid (152 Da), p-hydroxybenzoic acid (138 Da), and proline diketopiperazine (194 Da). Moreover, the effect was even more enhanced when these molecules were tested in combination [14].

In the last decade, a defensin peptide, lucifensin, from the ES and body tissues (fat body, haemolymph, and gut), was firstly reported. It consisted of 40 amino acid residues and three intramolecular disulphide bridges. The authors surmised that lucifensin was that long-sought antimicrobial factor of the maggot ES [15]. Andersen et al. used molecular biology methods to detect the sequence of lucifensin and found it was active against a range of* Staphylococcus* and* Streptococcus* species. As a homologue to other dipteran defensins, lucifensin had a similar antibacterial activity against Gram-positive bacteria [16]. The expression of lucifensin was strongly stimulated in the fat body when it was exposed to* S. aureus* and* P. aeruginosa* in vitro. However, the antimicrobial activity of ES was not enhanced by pathogens from the environment [17]. In the same year, lucifensin II, different from that of lucifensin by only one amino acid residue, was found from hemolymph of the maggot [18]. As a kind of cationic antibacterial peptides, lucifensin and lucifensin II were believed to kill bacteria by forming ion channels or transmembrane pores, causing leakage of cytoplasmic components from bacterial cell membrane [18, 19]. In a pioneering work, lucilin, a 36-residue cecropin-like antimicrobial peptide, was identified as a partial genetic sequence in maggots [20]. GWLK-Lucilin-CPD-His8, the form of recombinant fusion protein with a cysteine protease domain, was active against multidrug resistance Gram-negative bacteria and without hemolytic activity towards human erythrocytes.

Zhang et al. isolated and purified an antibacterial protein (<10 kDa) from maggots, named antibacterial protein from maggots (MAMP), which demonstrated inhibitory activity against both standard strains and clinically isolated antibiotic-resistant strains of* S. aureus*. Through scanning electron and transmission electron microscopy, the authors supposed the possible mechanism was the interaction with the bacterial cell membrane and disruption of the cell surface structure, leading to the increase of membrane permeability [21].

## 3. Antibiofilm Activity

Maggots also fight bacteria in their more resistant form: biofilm, which is a structured community living closely in a protective and self-produced polymeric matrix. It is widely accepted that biofilm associated infections are notoriously difficult to treat, even with advanced antibiotics.

More recently, a study demonstrated that maggot ES were differentially effective against biofilms of* S. aureus* and* p. aeruginosa*. It was found that ES could disrupt biofilm formation of* S. aureus* at a very low concentration of 0.2 *μ*g and rapidly degrade biofilms with the concentration increasing to 2 *μ*g. The collapse of* p. aeruginosa* biofilm required a high concentration of 20 *μ*g [22].

But what makes more sense is that maggot ES may act selectively against different bacterial strain. In the study of Bohova et al., ES were effective in the reduction of biofilm formation and the eradication of established biofilms of* S. aureus* and* E. cloacae*. On the contrary, ES stimulated* P. mirabilis *biofilm formation surprisingly [23]. They also found maggot ES eradicate the bacterial biofilm of different bacterial strains through different mechanisms. In the case of* E. cloacae*, preformed biofilm and viable cells were all disrupted. However, ES only prevented biofilm formation in terms of* S. aureus*. This was consistent with the previous observations [7, 24].

Cazander et al. found that maggot ES prevent and inhibit* P. aeruginosa* biofilm formation. The bioactivity of ES was still stable for 1 month at room temperature [25]. Moreover, in another study [26], the authors investigated the biofilm formation of other bacterial species (*S. aureus, S. epidermidis, K. oxytoca, E. faecalis*, and* E. cloacae*) on several biomaterials (polyethylene, titanium, and stainless steel). The observations suggested maggot ES could decrease biofilm formation and provide an innovative treatment for biofilm formation on infected biomaterials.

The specific mechanism was unclear; therefore, Harris et al. explored the interaction of ES and biofilm formation using two strains of* S. epidermidis* (1457 and 5179-R1). They found that mechanisms involved in biofilm formation were through the degradation of the polysaccharide intercellular adhesin (PIA) and accumulation associated protein (AAP), respectively. Inhibitory activity was sensitive to heat treatment, and the particular responsible molecule was in the >10 kDa fraction, appearing to have protease or glucosaminidase activity [24].

In another investigation, chymotrypsin 1 derived from maggot ES was found to be responsible for digesting collagens, which could be conducive to impede bacterial colonisation and subsequent biofilm formation [27]. After that, Harris et al. became interested in the recombinant chymotrypsin (rChymotrypsin) isolated from maggot ES. They studied the potential of this rChymotrypsin when it interfered with staphylococcal biofilms in the way similar to the above experiment. Compared with* S. epidermidis* 1457 and* S. aureus* SA113, the greatest effect of rChymotrypsin on both biofilms was seen on* S. epidermidis* 5179-R1, which has suggested that maggot rChymotrypsin disrupted protein adhesin-mediated biofilm formation of* Staphylococcus* [28].

Brown et al. purified a native molecular mass from the maggot ES, which was demonstrated to have a deoxyribonuclease (DNAse) activity. This maggot DNAse, approximately 45 kDa with magnesium, sodium, and calcium metal ion dependency, degraded extracellular bacterial DNA in biofilms preformed from* P. aeruginosa*. In addition, the DNAse was able to digest the DNA found in slough/eschar [29]. From an in vitro experiment, Cowan et al. demonstrated that maggot was effective in eliminating mature* S. aureus* biofilm from experimental pigskin explants within 24 hours [30].

## 4. Synergistic Effect with Antibiotics

Clinically, a combination of maggot ES with existing medical treatments may possess greater therapeutic potential. Therefore, van der Plas et al. performed a study to assess the effect of combination of maggot ES and antibiotics (vancomycin, daptomycin, and clindamycin) on* S. aureus* biofilms. It was suggested that the combination of maggot ES and antibiotics could break down* S. aureus* biofilms thus allowing the bacterial cells exposure to the antibiotics. They indicated that responsible molecules in maggot ES were proteases belonging to the group of serine proteases [31].

Then, a similar study was investigated whether maggot ES influence the antibacterial activity of different antimicrobial agents. The result showed a dose-dependent increase of the antibacterial effect of gentamicin and flucloxacillin in the presence of ES on* S. aureus *[32]. The combination effect of ES and ciprofloxacin also showed enhanced antibacterial activity against* S. aureus* at subinhibitory concentration [33].

## 5. Antifungal Bioactivity

So many studies have sought to determine the antibacterial components present in maggot ES. However, less well known are the literatures with regard to the antifungal properties.

As recorded by Alnaimat et al., the maggot ES contained a lot of alkaline compounds (e.g., ammonium carbonate, allantoin), which showed moderate antifungal activity against yeast and mould [34]. Pöppel et al. reported a novel antifungal peptide from the maggot, named lucimycin. The molecular weight of this peptide was 8.2 kDa with 77 amino acid residues [35]. Lucimycin was active against fungi from the* Ascomycota, Basidiomycota*, and* Zygomycota*, to the* Phytophthora parasitica*, but it was inactive against bacteria. The authors suggested that the lucimycin was likely to bind zinc and probably other divalent metal ions. Thus the mechanism was involved with not only the overall physicochemical properties of the peptide but also the metal complex formation to a specific receptor. In 2015, a paper published by Evans et al. demonstrated that maggot ES were active against* Candida albicans* and appeared to be highly heat stable and lyophilization resistant. The antifungal components were isolated in three fraction masses, >10, 10–0.5, and <0.5 kDa. The strongest level of activity was seen in the <0.5 kDa fraction [36].

Because of the lack of further experiments, the mechanism of antifungal activity is still unclear, but it probably differs from the antimicrobial mechanism of classical cationic AMPs. Hence, more efforts should be put to identify and chemically define the antifungal components. Meanwhile, a greater range of fungal pathogens and minimum inhibitory concentration testing should be carried out.

## 6. Anti-Inflammatory Activity

Inflammation is the first stage of wound healing. However, excessive inflammatory reaction is blamed for chronic wounds. Neutrophils, monocytes, and macrophages are main producers of ROS along with kinds of proinflammatory cytokines, therefore preventing the wound moving forward healing process.

The maggot ES was discovered to significantly reduce the level of both superoxide generation and myeloperoxidase (MPO) released from stimulated neutrophils rather than unstimulated neutrophils [37]. After that, van der Plas et al. assessed the effects of maggot ES on human neutrophils, monocytes, and macrophage in three associated experiments. In the first one [38], H_2_O_2_ production via fMLP- and PMA-activated neutrophils was found to be inhibited by maggot ES in a dose-dependent manner. Meanwhile, maggot ES reduced fMLP-stimulated expression of CD11b/CD18 and inhibited neutrophil chemotaxis towards fMLP. The authors suggested that maggot ES inhibit the proinflammatory responses of human neutrophils through a cyclic AMP-dependent mechanism. To confirm the supposition, they carried out the second experiment [39] and found that proinflammatory cytokines (IL-12p40, TNF-*α*, and MIF) from lipopolysaccharides-stimulated monocytes were decreased by maggot ES, whereas the anti-inflammatory cytokine IL-10 was enhanced. Finally, they drew the conclusion that maggot ES inhibited the proinflammatory responses of human neutrophils and monocytes through elevation of cyclic AMP. Moreover, maggot ES also decreased the chemotactic response of monocytes to fMLP, which coincided with the earlier conclusion of reducing the migration of human neutrophils towards fMLP. Of note, maggot ES did not affect the phagocytosis and intracellular killing of* Staphylococcus aureus*/*Candida albicans* by monocytes or neutrophils [38, 39]. According to third experiment [40], it was shown that monocyte-macrophage differentiation was altered by maggot ES from a proinflammatory to a proangiogenic type in the presence of maggot ES. Additionally, MØ-1 and MØ-2 were regulated by maggot ES with an increased production of MCP-1 and IL-8 and a reduced production of the chemokines MIP-1b, RANTES, and PDGF-BB.

In 2012, Cazander et al. firstly investigated the effect of maggot ES on complement activation (CA) in sera obtained from patients preoperatively and postoperatively. Maggot ES clearly reduced CA via all pathways, underlying the possible mechanism of breaking down complement proteins C3 and C4 in a cation-independent manner. The responsible ES component was supposed to be thermostable and has not been clearly identified [41]. In a further study, it was confirmed that maggot ES inhibited complement activation by two different mechanisms and downregulated the C3a/C5a-mediated neutrophil activation [42].

## 7. Immunomodulatory Function

In fact, immune modulation by maggot is a protective behavior in the field of MDT. Elkington et al. characterized a 56 kDa protein, blowfly larval immunosuppressive protein (BLIP), from maggot ES and identified it as a member of the serpin protein family. The authors found that maggot ES were capable of inhibiting mitogen-induced ovine T lymphocyte proliferation by decreasing expression of the activation marker CD25. Subsequently, the expression of the cytokine genes (IFN-*γ*, IL-4, IL-10, and IL-13) was downregulated. Contrarily, TNF-*α* and TGF-*β* gene expressions were upregulated in the presence of the BLIP. It was believed that the BLIP acted as an important immune evasion role to inhibit T lymphocyte activation when encountering a stimulus [43]. Zhang et al. also found that the crude extracts from maggots could raise the level of serum hemolysin and the data of carbon expurgatory test in a murine model [44].

## 8. Proangiogenic Activity

Healing of inflammation often involves growth of granulation tissue, which is composed mainly of capillaries and fibroblasts. Both laboratory and clinical studies about maggot-induced wound healing revealed profound angiogenesis [45].

The research from Wang et al. supported the notion that maggot ES promoted angiogenesis directly by inducing human microvascular endothelial cells (HMEC-1) migration and this event was partially mediated by the activation of AKT1, but not ERK1/2. This wound healing assay also indicated that maggot ES improved cell migration activity rather than increasing cell proliferation [46].

van der Plas et al. found that maggot ES enhanced the production of proangiogenic growth factors, such as vascular endothelial growth factor (VEGF) and basic fibroblast growth factor (bFGF) by macrophages [40]. Afterwards, three amino acids (histidine, valinol, and 3-guanidinopropionic acid) were identified within maggot ES and specifically exhibited significant proangiogenic effect on human umbilical vein endothelial cells (HUVECs), whilst having no effect on fibroblasts [47]. The result was confirmed by Sun et al. who found that maggot ES promoted HUVEC proliferation and increased expression of VEGF receptor 2. Moreover, a significant elevated level of CD34 and CD68 was also observed, which was indicative of promoting angiogenesis [48]. As well, another component extracted from maggot, fatty acid, was found to be favor of stimulating angiogenesis in a murine wound model via increasing VEGF expression (VEGFA mRNA and VEGFA protein expression) and wound capillary density. From the analysis of GC/MS, 80% of the extracts were unsaturated and probably were active ingredients [49].

Honda et al. reported a significant increase in endogenous hepatocyte growth factor (HGF) from blood samples of patients treated with MDT. The authors suggested that ES promoted HGF production via a positive feedback loop of HGF--c-MET--STAT3 and then promoted healthy granulation tissue [50]. The STAT3 signaling pathway was confirmed by Li et al. in a recent study. The authors detected five signaling pathways (Wnt2-, NF-*κ*B-, Notch1-, STAT3-, and TGF-*β*/Smad3), which are involved in wound healing and suggested that two signaling pathways (STAT3 and TGF-*β*/Smad3) were activated in the presence of maggot extracts, accompanied with their downstream gene expression (c-Myc, VEGF, and cyclin D1) [51].

## 9. Fibroblast Migration/Proliferation and ECM Remodelling

All kinds of trauma could cause different degrees of degeneration, necrosis, and tissue defect; therefore cell proliferation and EMC remodelling are prerequisite for tissue repair. Fibroblasts migrate from the edge of the wound and are crucial to the formation of granulation for reconstructing the wound defects. Fibroblasts also secrete proteases (e.g., serine proteases and matrix metalloproteinases) which are responsible for reorganization of the components within ECM.

In 1997, Prete firstly demonstrated that maggot ES/hemolymph stimulated the proliferation of fibroblasts and did not merely shift the kinetics of fibroblast growth. Furthermore, in the presence of epidermal growth factor (EGF), maggot ES/hemolymph significantly caused additional fibroplasia, suggesting a different mechanism to that of EGF, or a synergistic effect [52]. Recently, Smith et al. found that maggot ES markedly accelerated the migration of fibroblasts and epidermal keratinocytes during wound closure without any significant mitogenic effect [53].

The study by Horobin et al. confirmed the role of maggot ES proteinases, mainly serine proteinases, in promoting fibroblast migration. The authors pointed out that maggot ES promoted human dermal neonatal fibroblast (HDNF) cell migration, which was correlated to the degradation of fibronectin upon a fibronectin-coated surface [54]. In order to elucidate the mechanism behind the effect, the same authors developed a three-dimensional model to observe fibroblast migration and morphology in response to maggot ES. This novel model, which more closely mimicked the microenvironment* in vivo*, provided a much better understanding of the interactions between the ECM, resident cells, and maggot ES in the wound healing process. Possible mechanisms may be through the promotion of fibroblast motility, release of bioactive compounds from the ECM, and coordination of cellular responses [55]. Polakovičova et al. used an in vitro model similar to that of Horobin to observe the morphologic properties of fibroblasts exposed to maggot ES. The authors observed that, after 5 and 10 days of cultivation with maggot ES, there was an increased cell metabolism and protein production (secondary lysosomes and residual bodies) to produce the microfibrillar network which was necessary for their migration and the remodelling of the ECM [56].

Chambers et al. made a systematic survey of the proteolytic activity of maggot ES against physiologically relevant ECM components in vitro [57]. Three classes of proteolytic enzyme were detected in the maggot ES, including metalloproteinases, aspartyl proteases, and serine of two different subclasses (trypsin-like and chymotrypsin-like). Chymotrypsin-like serine proteinases were predominant to solubilize fibrin clots and degrade ECM components, such as fibronectin, laminin, and acid-solubilized collagen types I and III. The trypsin-like activity appeared to be crucial to PAR-mediated activation of proliferation or cytokine secretion within the wound. Following this investigation, a chymotrypsinogen from the maggot has been successfully cloned [58]. Compared with the chymotrypsins from bovine and human sources, the active recombinant chymotrypsin I was superior to degrade eschar ex vivo, which indicated a better proteolytic activity. The inhibition profile of the recombinant chymotrypsin I in comparison with human *α*-chymotrypsin was detected in another study [59]. The recombinant maggot chymotrypsin I still remained active within wound eschar; however, human *α*-chymotrypsin was inhibited by endogenous *α*1-antichymotrypsin and *α*1-antitrypsin.

## 10. Procoagulant Activity

The influence of maggot on blood coagulation has not been studied in detail so far. Until 2015, kahl et al. firstly identified and characterized the procoagulant properties of maggot ES [60]. The specific (chymo-) trypsin-like serine proteases induced clotting of human plasma and whole blood, particularly by activating contact phase proteins factors XII, IX, and kininogen. There was no obvious influence on platelet activation or fibrinolysis. More recently, the authors separated the maggot ES and analysed their components, resulting in the identification of a chymotrypsin-like serine protease, Jonah-like protein. This protein was shown to reduce the clotting time of human plasma, even in the absence of the endogenous protease kallikrein, factors XI, XII [61].

## 11. Neuranagenesis

Zhang et al. found that the homogenate products from disinfected maggot increased protein gene product 9.5 (PGP 9.5) expression and substance P secretion in the murine cutaneous wound model [62]. PGP 9.5 is localised in normal neural and neuroendocrine tissues [68]. Substance P is mainly present in sensory nerve endings of skin tissue and has neurotransmitter and neuromodulatory effects [69]. Thus, the authors hypothesized that maggot homogenate products could promote wound nerve regeneration and neuropeptides release. Recently, Chu and Yan demonstrated that PGP 9.5 was highly expressed in wounds treated with maggots, compared with mupirocin [63]. However, there was still a lack of enough basic research in the field of neuranagenesis.

## 12. Antitumor Activity

The fatty acids (FA1 and FA2) extracted from maggot showed remarkable inhibitory activities against human leukemia cells HL-60 and human lung cancer cells A-549 in vitro [64]. The main active component of the fatty acids was polyunsaturated fatty acid (PUFA), especially *ω*-6 PUFA. Zhang et al. established a H22 hepatoma-bearing mice model and discovered that maggot ES could inhibit H22 tumor growth possibly through the activation of p38 mitogen-activated protein kinase (p38MAPK) signal pathway [65].

## 13. Antiatherosclerosis Activity

In an atherosclerosis mice model, maggot ES showed certain antiatherosclerosis effect that could decrease the serum level of triglyceride (TG), total cholesterol (TC), and low density lipoprotein (LDL) and increase the level of high density lipoprotein (HDL) [66]. Moreover, it could effectively regulate proliferation, differentiation, and secretory function of lymphocytes along with CD4+/CD8+ ratio in atherosclerosis, which may be one of the mechanisms of antiatherosclerosis activity [67].

## 14. Discussion

Many people may have their doubts about using maggots as medicine. However, they should keep in mind that a large number of drugs come from nature's pharmacy. For example, hirudin, a bivalent direct thrombin inhibitor extracted from leeches, is approved for heparin-induced thrombocytopenia and acute coronary syndromes [70]. In addition, anti-inflammatory protein-2, a protein secreted by hookworms, was found to serve as a novel curative therapeutic for allergic asthma and potentially other inflammatory diseases [71].

Similarly, maggot is also a real treasure-trove of raw materials for clinical application. MDT was used extensively in hospitals during the 1930s and 1940s. The application of MDT nearly disappeared from the early 1940s to 1980s because of the invention of penicillin and better surgical techniques. However, with the dilemma of antibiotic resistance and a diminishing arsenal of effective antibiotics, MDT revived as a potent, universal therapy for chronic, intractable wounds infected with MRSA and has been proven effective in cleaning and healing wounds that respond poorly to other forms of conventional treatments. Even more surprising is that the combinations of MDT and antibiotics enhance antibacterial activity. In addition, maggot produces a variety of active ingredients, such as lucifensin with antibacterial activity, lucimycin with antifungal bioactivity, and chymotrypsin with proteolytic activity. For convenience, relevant activities and responsible components/mechanisms within maggots are summarized in Table 1. Nonetheless, many other molecules with beneficial activities still need to be further identified and characterized. For this purpose, next generation sequencing could be available for researchers to focus their attention on genomic information. For example, Franta et al. applied next generation sequencing to analyze the transcriptome of maggots and identified 577 clusters representing 185 peptidase species [72]. Although detailed function of these putative peptidases was not provided, it still produced enough data for the further identification of bioactive proteins within maggots.

All these molecules could be potential candidates for therapy, and hence high purification and mass production are top priority for wide use. As is known to all, it is almost impossible to obtain a large number of highly purified proteins from original organic sources. Recombinant protein technology has quite acceptably solved this problem in several researches. Recently, the use of transient viral-based and transgenic systems to express heterologous proteins in insect larvae has attracted much attention. For example, Fossgreen et al. utilized transgenic system to produce human amyloid precursor protein, a primarily nonsecreted protein, in* Drosophila melanogaster *[73]. More similar to the aforementioned study, Linger et al. have created and characterized strains of transgenic maggot that express and secrete human platelet derived growth factor-BB (PDGF-BB) [74]. PDGF-BB has been investigated as a possible treatment for nonhealing wounds by stimulation of cell proliferation and survival, production and secretion of growth factors, and ECM constituents [75]. This research demonstrated for the first time that the maggot was capable of producing a human growth factor from the transgene expression systems. It sheds a light on the combination of MDT and genetic engineering to a new clinical application. Predictably, genetically modified maggot will be engineered to express and secrete diverse heterologous proteins which are more beneficial to wound healing in the future.

## 15. Conclusion

The “medicinal maggot,” which is the core of MDT, demonstrates possessing diverse properties. We summarize the current scientific laboratory evidences of the antimicrobial, antibiofilm, anti-inflammatory, and wound healing activities for MDT, particularly active ingredients (e.g., lucifensin, Seraticin, Chymotrypsin, DNAse, and unsaturated fatty acid) within maggots involved in these beneficial effects. The developments of recombinant and transgenic bioactive molecules from the maggot are both challenges and opportunities for increased treatment acceptance and improved outcome. Perhaps one day in the future, these molecules may surpass the use of the maggot itself in the treatment of chronic and refractory wounds. Sincerely, we hope to form a more detailed theory foundation from which great efforts towards therapeutic agents of maggots for medical purposes can be made.

## Figures and Tables

**Table 1:** Overview of the bioactivities of maggot.

Bioactivity	Substance/component/molecule	Object	Mechanism	Reference
Antibacterial	Ammonium carbonate Allantoin	-	Raise PH value	[10]
	Lucifensin	*Staphylococcus Streptococcus* species	Form ion channels or transmembrane pores	[15]
	Lucifensin II	*Staphylococcus aureus*, *Pseudomonas aeruginosa*	-	[18]
	Lucilin	Multidrug resistance Gram-negative bacteria	-	[20]
	MAMP	Standard and antibiotic-resistant strains of *Staphylococcus aureus*	Increase membrane permeability	[21]
	Seraticin	*Bacillus cereus Escherichia coli*	Lysis of the bacterial membrane	[13]
	p-Hydroxyphenylacetic acid p-Hydroxybenzoic acid Proline diketopiperazine	*Micrococcus luteus, Pseudomonas aeruginosa*	-	[14]

Antibiofilm	Chymotrypsin 1	*Staphylococcus aureus Staphylococcus epidermidis*	Disrupt protein adhesin-mediated biofilm formation	[27]
	DNAse	*Pseudomonas aeruginosa*	Digest DNA in biofilm	[29]

Synergistic effect with antibiotic	Serine proteases	Gentamicin, flucloxacillin Ciprofloxacin Vancomycin, daptomycin, clindamycin	Reduce minimal inhibitory concentration (MIC) Break down biofilm to allow the bacterial cells exposure to the antibiotics	[31–33]

Antifungal	Lucimycin	*Ascomycota, Basidiomycota, Zygomycota, Candida albicans, * *Phytophthora parasitica*	Metal complex formation to a specific receptor	[35, 36]

Anti-inflammation	ES	Neutrophils	Reduce superoxide generation, myeloperoxidase, and CD11b/CD18 Inhibit neutrophil chemotaxis Cyclic AMP-dependent mechanism	[38]
	ES	Monocytes	Inhibit monocytes chemotaxis Decrease in IL-12p40, TNF-*α*, MIF Increase in IL-10	[39]
	ES	Macrophage	Differentiate from proinflammatory to proangiogenic type Decrease MIP-1*β*, RANTES, and PDGF-BB Increase MCP-1 and IL-8	[40]
	Kind of thermostable protein	Complement system	Break down complement proteins C3 and C4	[41, 42]

Immunomodulatory	BLIP	T lymphocyte	Downregulate IFN-*γ*, IL-4, IL-10, IL-13, and CD25 Upregulate TNF-*α* and TGF-*β*	[43]

Proangiogenic	Unsaturated fatty acid	Microvascular endothelial cell	Improve migration Activate AKT1 signaling pathway	[49]
	Histidine Valinol 3-guanidinopropionic acid	Human umbilical vein endothelial cell	Improve migration Enhance VEGFR-2, CD34 and CD68	[47]
	Not identified	Hepatocyte growth factor	Downregulate c-Myc, VEGF, and cyclin D1 Activate STAT3 and TGF-*β*/Smad3 signaling pathway	[50, 51]

Fibroblast migration/proliferation and ECM remodelling	Kind of serine proteinases	Fibroblasts keratinocytes	Increase cell metabolism and protein production (secondary lysosomes and residual bodies)	[54, 55]
	Trypsin/chymotrypsin-like serine Metalloproteinases Aspartyl proteases	EMC components	Degrade fibronectin Solubilize fibrin clots Digest ECM components	[57]

Procoagulant	Jonah-like protein	Human plasma Whole blood	Reduce clotting time Activate contact proteins factors XII, IX, and kininogen	[61]

Neuranagenesis	Homogenate products	-	Promote neuropeptides (PGP 9.5 and substance P) release	[62, 63]

Antitumor	*ω*-6 PUFA	Human leukemia cells A-549 lung cancer cells H22 hepatoma	Activate p38MAPK signal pathway	[64, 65]

Antiatherosclerosis	ES	Murine serum	Decrease TC, TG, LDL Increase HDL Regulate CD4+/CD8+ ratio	[66, 67]
